# Interleukin-1 Mediates Neuroinflammatory Changes Associated With Diet-Induced Atherosclerosis

**DOI:** 10.1161/JAHA.112.002006

**Published:** 2012-06-22

**Authors:** Adam Denes, Caroline Drake, Jing Stordy, Janet Chamberlain, Barry W. McColl, Hermann Gram, David Crossman, Sheila Francis, Stuart M. Allan, Nancy J. Rothwell

**Affiliations:** Faculty of Life Sciences, University of Manchester, Manchester, UK (A.D., C.D., S.M.A., N.J.R.); Department of Cardiovascular Science, Medical School, University of Sheffield, Sheffield, UK (J.S., J.C., D.C., S.F.); The Roslin Institute and R(D)SVS, University of Edinburgh, UK (B.W.M.); Norwich Medical School, University of East Anglia, Norwich, UK (D.C.); Novartis Institutes of BioMedical Research, Basel, Switzerland (H.G.)

**Keywords:** interleukin-1, atherosclerosis, cerebrovascular inflammation, leukocyte, microglia

## Abstract

**Background:**

Systemic inflammation contributes to brain pathology in cerebrovascular disease through mechanisms that are poorly understood.

**Methods and Results:**

Here we show that atherosclerosis, a major systemic inflammatory disease, is associated with severe cerebrovascular inflammation in mice and that this effect is mediated by the proinflammatory cytokine interleukin-1 (IL-1). Apolipoprotein E–deficient mice fed Paigen or Western diets develop vascular inflammation, microglial activation, and leukocyte recruitment in the brain, which are absent in apolipoprotein E–deficient mice crossed with IL-1 type 1 receptor–deficient mice. Systemic neutralization of IL-1β with an anti–IL-1β antibody reversed aortic plaque formation (by 34% after a Paigen and 45% after a Western diet) and reduced inflammatory cytokine expression in peripheral organs. Central, lipid accumulation–associated leukocyte infiltration into the choroid plexus was reversed by IL-1β antibody administration. Animals fed a Western diet showed 57% lower vascular inflammation in the brain than that of mice fed a Paigen diet, and this was reduced further by 24% after IL-1β antibody administration.

**Conclusions:**

These results indicate that IL-1 is a key driver of systemically mediated cerebrovascular inflammation and that interventions against IL-1β could be therapeutically useful in atherosclerosis, dementia, or stroke. **(*J Am Heart Assoc.* 2012;1:e002006 doi: 10.1161/JAHA.112.002006.)**

## Introduction

Inflammation is a key driver of cardiovascular and cerebrovascular disease, including atherosclerosis, and contributes to diseases such as myocardial infarction, stroke, and dementia. These diseases share common risk factors, such as obesity, hypertension, and diabetes,^1,2^ that are associated with inflammation. In patients, a clear association exists between cardiovascular risk factors or carotid atherosclerosis and dementia, progression of Alzheimer's disease, or depression.^3–5^ Animal studies demonstrate that peripheral inflammatory conditions are associated with increased neuroinflammatory responses in the brain and enhanced damage in response to experimentally induced stroke.^6–9^

Interleukin (IL)-1 is a key contributor to a diverse range of diseases, demonstrated by the beneficial effects of blocking endogenous IL-1 in experimental models, most commonly with the IL-1 receptor antagonist (IL-1Ra).^10^ Evidence indicates that IL-1 contributes to peripherally induced neuroinflammatory diseases. Mice that lack IL-1 type 1 receptor (IL-1R1), when subjected to experimental autoimmune encephalomyelitis, show significantly reduced neuroinflammation, as indicated by a reduction in vascular adhesion molecule (VCAM)-1 and infiltrating immune cells.^11^ IL-1 expression is increased in human coronary arteries affected by atherosclerosis^12,13^ and is also associated with arterial inflammation, oxidative stress, and increased blood pressure.^14^

In apolipoprotein E–knockout (ApoE^−/−^) mice fed high-fat diets, atheroma development is related to the composition of the diet; a high-fat, high-cholate (Paigen) diet produces the largest effect.^14^ Genetic deletion of IL-1R1 in ApoE^−/−^, Paigen diet–fed mice (ApoE^−/−^/IL-1R1^−/−^) markedly reduces inflammatory responses in the periphery.^14^ Similar results were seen in Paigen diet–fed ApoE^−/−^ mice treated with a neutralizing antibody to IL-1β.^15^ These findings suggest that IL-1 mediates the effects of high-fat diets on peripheral vascular atherosclerotic pathology.

Our recent studies show increased cerebrovascular and brain inflammation in ApoE^−/−^ mice fed an atherogenic high-fat diet, though the mechanisms of this response are not known.^6^ The primary objective of the present study was to test the hypothesis that the neuroinflammatory responses associated with atherosclerosis induced by high-fat feeding in susceptible (ApoE^−/−^) mice is mediated by IL-1. To address this, we determined the neuroinflammatory response to fat feeding in ApoE^−/−^/IL-1R1^−/−^ mice and the effect of immunoneutralization of IL-1β on peripheral and central pathology in atherogenic mice.

## Methods

### Animals

Experiments were carried out in male ApoE^−/−^ (JAX 2052, Jackson Laboratories, Bar Harbor, Maine, USA) and ApoE^−/−^/IL-1R1^−/−^ mice, the latter generated as described previously.^14^ Both strains were bred in house at the University of Sheffield, UK, were allowed free access to food and water, and were maintained under temperature-, humidity-, and light-controlled conditions. All animal procedures adhered to the UK Animals (Scientific Procedures) Act (1986).

Ten-week-old mice were fed a control (4.3% fat, 0.02% cholesterol), Paigen (18.5% fat, 0.9% cholesterol, 0.5% cholate, 0.26% sodium), or Western (21% fat, 0.15% cholesterol, 0.03% cholate, 0.296% sodium) diet for a period of 8 weeks, as described previously.^14^ Body weight was recorded weekly, and, as a measure of well-being and overall fitness, voluntary wheel running activity was assessed. Blood pressures were recorded weekly by using a Visitech tail cuff system, as previously described.^14^ At the end of the experimental period, blood was taken from the heart, before transcardial perfusion. Brain, liver, and spleen samples were taken and prepared as described previously,^6,16^ and the aortae were fixed and harvested for en face Oil Red O staining and aortic root analysis.^14^

### Treatment

Mice were dosed with an anti-mouse anti–IL-1β or anti–cyclosporin A isotype control antibody^17,18^ according to weight (10 mg/kg IP).

### Cytokine Measurements

Eleven cytokines were measured in plasma (at weeks 0, 4, and 8), liver homogenates, and spleen homogenates: tumor necrosis factor-α, RANTES (CCL5), monocyte chemoattractant protein-1 (CCL2), KC (CXCL1), IL-6, IL-1β, IL-1α, IL-17α, interferon-γ, granulocyte-colony stimulating factor, and IL-10. These were measured with appropriate Cytometric Bead Array Flex Sets (BD Biosciences, UK) according to the manufacturer's protocol. Protein concentrations were calculated by bicinchoninic acid assay (Pierce/Thermo Fisher Scientific, UK).

### Assessment of Vascular and Microglial Activation by Immunohistochemistry

Immunohistochemistry for vascular (VCAM) and microglial ionized calcium binding adaptor molecule 1 (Iba1) activation was performed on free-floating brain sections. Endogenous peroxidase activity and blocking treatment were performed as stated previously. Primary antibody incubation was performed overnight with goat anti-mouse VCAM-1 1:250 (R&D Systems, Abingdon, UK) or rabbit anti-Iba1 1:1000 (Wako Chemicals, Nuss, Germany). Sections were then incubated in appropriate biotinylated secondary antibody for 1 hour (rabbit anti-goat 1:1000 and goat anti-rabbit 1:750, Vector Laboratories, UK), followed by Vectastain ABC solution (Vector Laboratories, UK), before visualization with nickel-enhanced diaminobenzidine (50 mg/mL) (Vector Laboratories, UK). Sections were mounted onto gelatin-coated slides and dehydrated, and coverslips were applied with Depex (Fisher, UK). Images were collected on an Axiocam color charge-coupled device camera (Zeiss, Germany) upright microscope with 10× and 20× objectives and were captured by a Coolsnap ES camera (Photometrics, Tucson, Arizona, USA) through Axiovision software (Zeiss, Germany).

### Assessment of Leukocyte Recruitment in the Brain

Double immunofluorescence was performed to detect leukocytes (CD45) and vascular activation (VCAM) on free-floating brain sections. After blocking in 2% normal donkey serum (Vector Laboratories, Burlingame, CA), sections were incubated overnight at 4°C with primary antibodies: rat anti-mouse CD45 1:200 (Serotec, UK) and goat anti-mouse VCAM-1 1:250 (R&D Systems). The antigens were visualized with the adequate fluorochrome-conjugated (Alexa 594 1:750 or Alexa 488 1:500, Molecular Probes, Eugene, OR) secondary donkey anti-sera for 2 hours at room temperature. Sections were mounted onto gelatin-coated slides and coverslipped with Vectashield mounting medium containing diamidinophenylindole (DAPI, Vector Laboratories, Burlingame, CA).

Images were collected on an Olympus BX51 upright and captured by a Coolsnap ES camera (Photometrics, UK) through MetaVue Software (Molecular Devices, UK).

### Histology

Oil Red O staining combined with CD45 immunohistochemistry was performed as described earlier.^6^

### Quantitative Analysis

All analyses were performed blinded. VCAM-positive blood vessels were counted in 3 random fields of view for each section (typically 8 to 10) containing rostro-caudal cerebral cortex. A score for the whole brain was obtained by averaging individual counts and was expressed as positive blood vessels per square millimeter. Activated microglia were identified as showing: (1) increased Iba1 immunopositivity, (2) enlarged or amoeboid cell body, and (3) complete or partial loss of thin, elongated processes. Round, small Iba1-positive cells with leukocyte morphology were not counted. Activated microglia were counted throughout the striatum and expressed as activated microglia per square millimeter. Fluorescently labeled CD45-positive cells were counted in the caudal choroid plexus (−1.82 mm from bregma) and the lateral ventricle (−1.58 mm from bregma). The choroid plexus and ventricular ependyma were visualized by VCAM immunofluorescence. Data on the control mice used to evaluate CD45, VCAM, and Iba1 staining in ApoE^−/−^/IL-R1^−/−^ have been published previously.^6^

### Statistical Analysis

Normal distribution of experimental data was examined by Shapiro-Wilk normality test. Only a minority of data sets did not pass the normality test (see Results), in which cases appropriate nonparametric tests (Mann-Whitney test for 2 groups and Kruskal-Wallis test followed by Dunn multiple-comparison test for ≥3 groups) also were performed. When normality of the data had been confirmed, comparisons between 2 experimental groups were made with unpaired *t* tests. Data from 4 groups (ApoE^−/−^/IL-R1^−/−^ experiment) were analyzed by one-way analysis of variance (ANOVA), followed by Bonferroni multiple-comparison post-test with GraphPad Prism 5 software. Two-way ANOVA was used to determine the overall effect of diet and IL-1β on the levels of vascular and microglial activation and recruitment of CD45-positive cells. Body weight changes were analyzed by repeated-measures ANOVA. Blood pressure measurements were analyzed by global nonlinear regression (to fit blood pressure data over a defined period of time for a group of animals), followed by an F test (GraphPad Prism 5). Data are presented as mean±SEM. A probability of <5% was regarded as statistically significant.

## Results

### Neuroinflammation in Atherosclerotic ApoE^−/−^ Mice Is Abolished in ApoE^−/−^/IL-R1^−/−^ Mice

**Figure 1.**
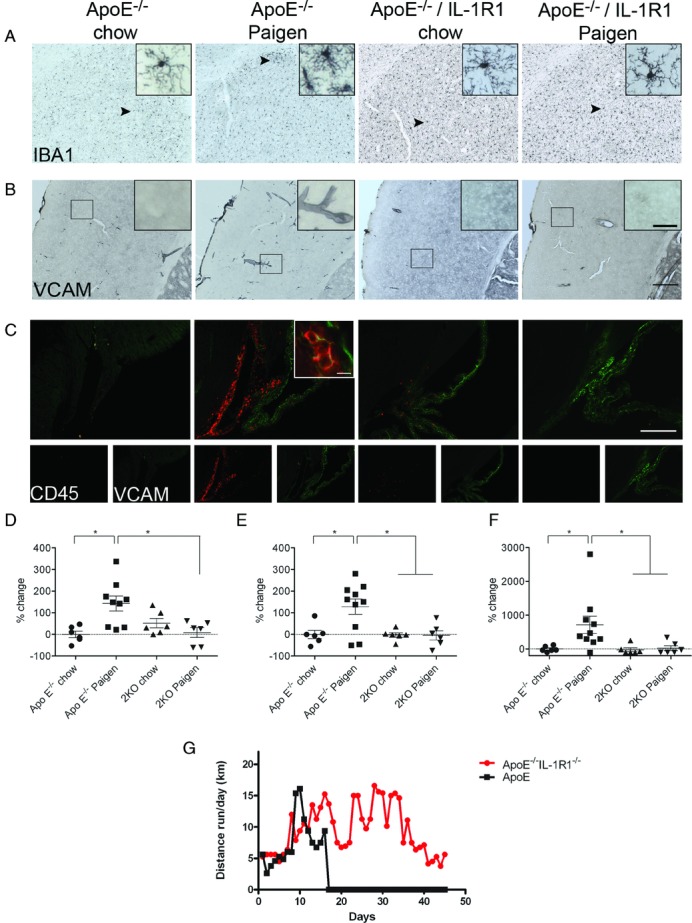
Vascular activation, microglial activation, and leukocyte accumulation are reduced in ApoE^−/−^/IL-1R1^−/−^ mice fed a Paigen diet. Activated microglia as identified by increased Iba1 immunopositivity, thickened processes, and irregular cell bodies were seen in ApoE^−/−^ mice fed a Paigen diet. Microglial activation was significantly reduced, to control levels, in ApoE^−/−^/IL-1R1^−/−^ mice fed a Paigen diet (A). Vascular activation was assessed through the immunostaining of the adhesion molecule VCAM. Atherosclerotic ApoE^−/−^ mice show increased vascular activation, which is significantly reduced in ApoE^−/−^/IL-1R1^−/−^ mice fed a Paigen diet (B). Leukocyte accumulation as shown by CD45 immunostaining was increased in ApoE^−/−^ mice fed a Paigen diet. Leukocyte accumulation was significantly reduced, to control levels, in ApoE^−/−^/IL-1R1^−/−^ mice fed a Paigen diet (C). D, Quantification of Iba1-positive microglia. E, Quantification of VCAM-positive blood vessels. F, Quantification of CD45-positive leukocytes. The “vehicle” data in F were not normally distributed; therefore, in addition to one-way ANOVA (*P*=0.017) followed by Bonferroni multiple-comparison post-test, nonparametric Kruskal-Wallis test (*P*=0.0065) followed by Dunn multiple-comparison test also were performed. Post-hoc comparisons gave identical results. A through F: n=6–10. G, Voluntary wheel running actogram for ApoE^−/−^ and ApoE^−/−^/IL-1R1^−/−^ mice (n=2–3). Error bars represent standard error, *P<0.05. Scale bars: 200 μm (A through C) and 10 μm (inset in C).

Diet-induced atherosclerosis in *ApoE^−/−^* mice was associated with marked neuroinflammatory responses in the brain, which were abolished in animals in which the IL-1 receptor was deleted. Specifically, in ApoE^−/−^/IL-R1^−/−^ mice fed a Paigen diet, microglial activation was significantly reduced compared to ApoE^−/−^ mice fed a Paigen diet and was similar to that of mice fed a control diet (Figure 1A and 1D). VCAM-1 was upregulated throughout the brain in ApoE^−/−^ mice fed a Paigen diet, whereas there was little or no evidence of vascular activation in ApoE^−/−^/IL-R1^−/−^ mice fed a Paigen diet, and in these animals vascular activation was comparable to that of mice fed a control diet and was significantly lower than in ApoE^−/−^ mice fed a Paigen diet (Figure 1B and 1E). The enhanced leukocyte accumulation in ApoE^−/−^ mice fed a Paigen diet was reversed in ApoE^−/−^/IL-R1^−/−^ mice to levels comparable to the mice fed a control diet (Figure 1C and 1F). The marked decline in voluntary physical activity in ApoE^−/−^ mice was also reversed by genetic deletion of the IL-1 receptor in the ApoE^−/−^/IL-1R1^−/−^ mice (Figure 1G).

### Neutralization of IL-1β Reduces Atherosclerotic Lesion Size in ApoE^−/−^ Mice Fed a High-Fat Diet

**Figure 2.**
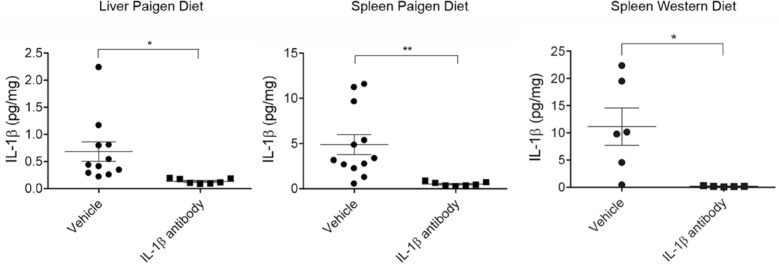
Neutralization of IL-1β in anti–IL-1β antibody–treated mice. Cytokines were measured by Cytometric Bead Array. IL-1β was neutralized in the livers and spleens of ApoE^−/−^ mice fed a Paigen diet and in the spleens of mice fed a Western diet, as compared with mice treated with control antibody. Error bars represent standard error, *P<0.05 and **P<0.01 vs ApoE^−/−^ mice treated with a control antibody (n=7–11).

Peripheral administration of an anti–IL-1β antibody successfully neutralized IL-1β (an average 89% reduction in IL-1 concentrations) in all organs where IL-1β expression was detectable, regardless of diet (Figure 2).

**Figure 3.**
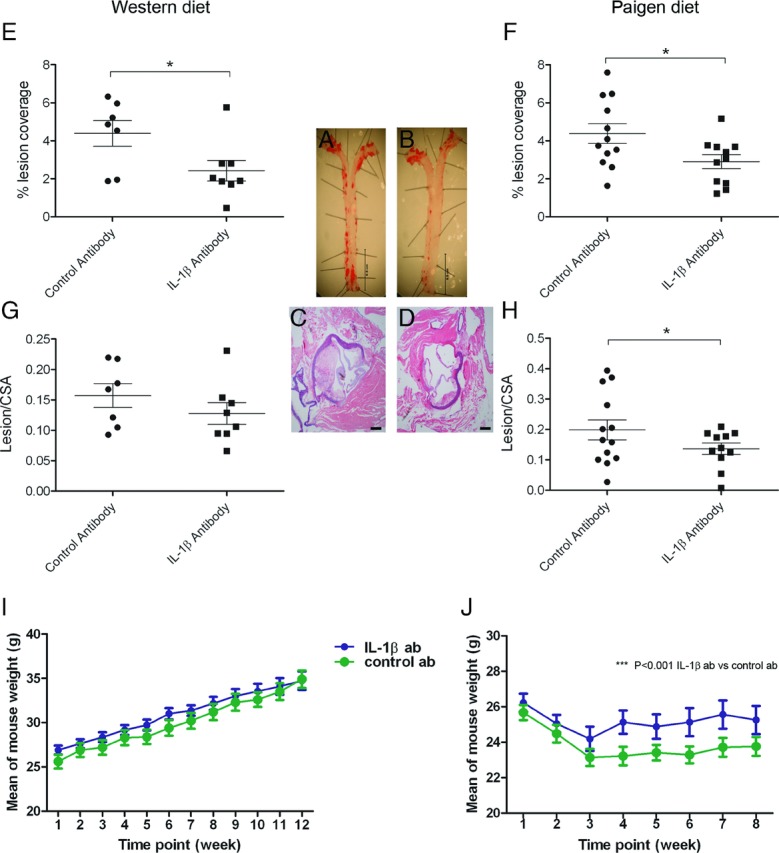
Atherosclerotic burden is reduced after treatment with an IL-1β antibody. Atherosclerosis was assessed by Oil Red O staining of whole aortae and histology of lesions at aortic roots. Inset (A through D), Representative histology from Paigen diet–fed mice. Whole aortae in A (control antibody) and B (IL-1β antibody), with en face Oil Red O staining; aortic root in C (control antibody) and D (IL-1β antibody), with Millers’ elastic van Gieson staining. Reduction in percent coverage of lesions in aortae from ApoE^−/−^ mice fed a Western (E) and Paigen diet (F) with and without antibody treatment (both *P<0.05). Aortic root lesion size in mice fed a Western (G) and Paigen (H) diet (*P<0.05) when treated with an IL-1β antibody. Body weight changes in mice fed a Western (I) or Paigen (J) diet over the course of the study. Scale bars: 0.5 mm (A and B) and 50 μm (C and D). n=7–8 for E and G; n=11–13 for F and H.

Lesion coverage in the descending aorta was reduced significantly in IL-1β antibody–treated mice fed either a Paigen or a Western diet, as compared to control mice (by 34% and 45% respectively; Figure 3A and 3B). Mice treated with IL-1β antibody had significantly smaller lesion areas after having been fed the Paigen diet, as compared to control antibody–treated mice (Figure 3D). There was no diet-dependent difference in lesion size or percentage coverage in either the IL-1β or control antibody treatment groups. Treatment with IL-1β antibody had no statistically significant effect on systemic blood pressure in mice fed either diet (data and statistical analysis are not shown).

IL-1 neutralization reversed the weight loss in mice fed a Paigen diet over the course of the study period (*P*<0.001), whereas there was no difference in the body weight of mice fed a Western diet, with or without intervention (Figure 1).

### IL-1β Neutralization Reduces Peripheral Inflammation

**Figure 4.**
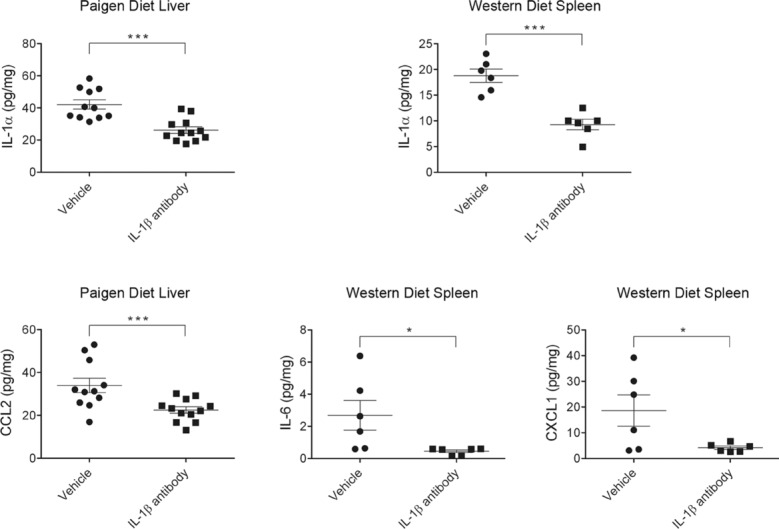
IL-1β neutralization has secondary antiinflammatory effects in peripheral organs. Cytometric Bead Array was used to measure the cytokine and chemokine levels in homogenized livers and spleen samples. IL-1α was significantly reduced in livers of ApoE^−/−^ mice fed a Paigen diet and the spleens of mice fed a Western diet. The chemokine CCL2 was reduced in the livers of ApoE^−/−^ mice fed a Paigen diet. The concentrations of interleukin-6 and CXCL1 were significantly reduced in response to IL-1β neutralization in mice fed a Western diet. Error bars represent standard error, *P<0.05, **P<0.01 and ***P<0.001 vs ApoE^−/−^ mice treated with a control antibody (n=6–12).

Consistent with the markedly reduced IL-1β levels described above, IL-1α was also reduced significantly (37%) in the livers of mice fed the Paigen diet and in the spleens of mice fed the Western diet (42%; Figure 4), but no significant reduction was seen in the spleens of mice fed a Paigen diet and in the livers of mice fed a Western diet. IL-1β neutralization also reduced hepatic concentrations of CCL2 (33%) in mice fed a Paigen diet and interleukin-6 (80%) and CXCL1 (79%) levels in spleens from mice fed a Western diet (Figure 4). No significant differences were observed in CCL2, interleukin-6, or CXCL1 levels in other organs in mice fed a Paigen or Western diet. Levels of IL-17, interferon-γ, IL-10, or tumor necrosis factor-α were mostly below the detection limit of the assay or the results were inconsistent; therefore, no significant effect of IL-1β neutralization was observed. The lack of statistical significance might be a result of underpowered analysis in some cases.

### IL-1β Neutralization Attenuates Vascular Activation in ApoE^−/−^ Mice Fed a Western Diet

**Figure 5.**
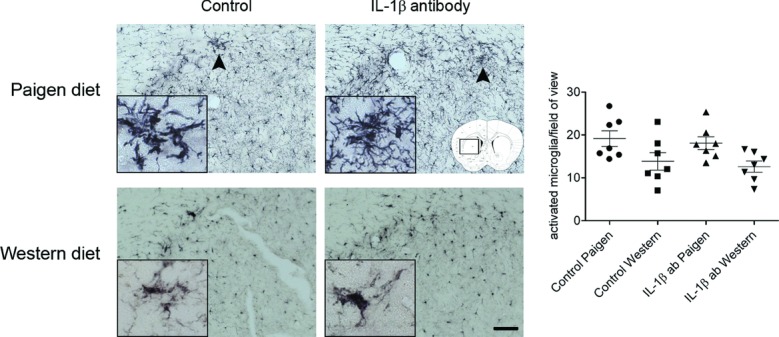
Levels of microglial activation differed between ApoE^−/−^ mice fed a Paigen or Western diet. Levels of microglial activation were assessed through the immunostaining of Iba1. Microglial activation was assessed throughout the striatum, and the location of images shown is indicated in the schematic diagram. Levels of microglial activation differed for each diet, but IL-1β neutralization had no effect. Error bars indicate standard error (n=7), scale bars 200 μm and 10 μm.

**Figure 6.**
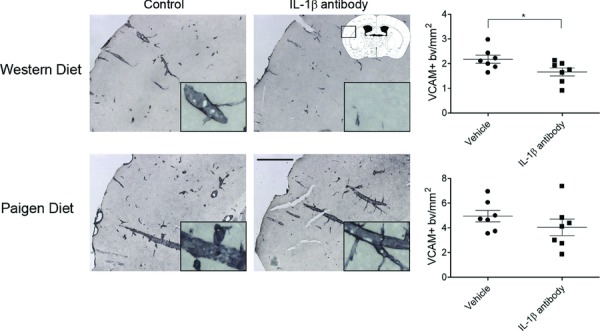
IL-1β neutralization reduces vascular activation in ApoE^−/−^ mice fed a Western diet. Levels of vascular activation were assessed with immunostaining for the adhesion molecule VCAM. Vascular activation was assessed throughout the cerebral cortex; locations of images shown are indicated in the schematic diagram. Vascular activation was significantly reduced in ApoE^−/−^ mice fed a Western diet compared to those fed a Paigen diet. IL-1β neutralization caused a significant reduction in vascular activation in ApoE^−/−^ mice fed a Western diet. Error bars indicate standard error, *P<0.05 vs ApoE^−/−^ fed a Western diet (n=7). Scale bars 200 μm and 50 μm.

Microglial and vascular activation have been reported previously in the brains of atherosclerotic ApoE^−/−^ mice fed a Paigen diet, but no information is available on mice fed a Western diet. Mice fed a Paigen diet showed microglial activation that was 41% higher than in mice fed a Western diet (Figure 5). Microglial activation was observed in the cerebral cortex, striatum, periventricular areas, and meninges, as reported previously.^6^ The number of activated microglial cells in mice fed a Paigen or Western diet was not different after IL-1β neutralization (Figure 5). Mice fed the cholate-free, high-fat, Western diet showed a 57% reduction in vascular activation when compared to mice fed a Paigen diet, as indicated by a lower number of VCAM-positive blood vessels (Figure 6B and 6C). IL-1β neutralization had no significant effect on vascular activation in ApoE^−/−^ mice fed a Paigen diet, although a trend toward reduced activation was observed (the lack of statistical significance might be a result of underpowered analysis). In contrast, the neutralization of IL-1β significantly reduced (24%) vascular activation in mice fed a Western diet (Figure 6B and 6C).

### IL-1β Neutralization Attenuates the Accumulation of CD45+ Leukocytes in ApoE^−/−^ Mice in the Lateral Ventricle

**Figure 7.**
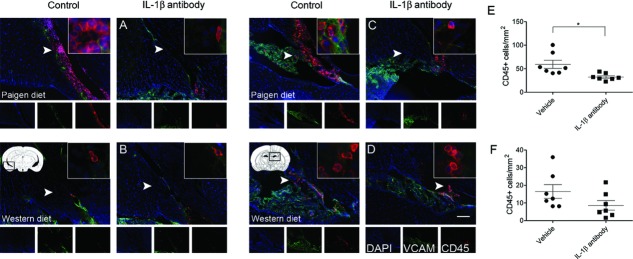
Leukocytes accumulate in the choroid plexus of the lateral ventricle in ApoE^−/−^ mice fed a Paigen diet and, to a lesser extent, a Western diet, which is attenuated in the Paigen diet–fed mice by an anti–IL-1β antibody. A, CD45-positive leukocytes accumulate in the ventral, caudal part of the lateral ventricle in ApoE^−/−^ mice fed a Paigen diet, which is attenuated by IL-1β antibody treatment. B, Accumulation of leukocytes in this region is not seen in mice fed a Western diet. C, CD45-positive leukocytes accumulate in the choroid plexus of the lateral ventricle in ApoE^−/−^ mice fed a Paigen diet, which is attenuated by IL-1β antibody treatment. Data in C were not normally distributed; therefore in addition to unpaired *t* test (*P*=0.013), nonparametric Mann-Whitney test (*P*=0.0041) was also performed. D, Fewer CD45-positive cells accumulated in this region in mice fed a Western diet, but IL-1β antibody treatment had no significant effect. Schematic diagram indicates the location of images. E and F, Quantification of the total number of CD45-positive leukocytes in mice fed a Paigen (E) or Western (F) diet. Error bars represent standard error, *P<0.05 vs ApoE^−/−^ mice treated with a control antibody (n=7).

Diet-dependent leukocyte accumulation was assessed in coronal brain sections and was found to occur in 2 different regions of the lateral ventricle (Figure 7A and 7D). Mice fed a Paigen diet showed increased leukocyte accumulation, principally in an area of the dorsal lateral ventricle between the hippocampus and the thalamus, as previously shown.^6^ Treatment with the anti–IL-1β antibody significantly (*P*<0.05) attenuated the number of leukocytes accumulating in the dorsal lateral ventricle in response to the Paigen diet (Figure 7A). In contrast, mice fed a Western diet showed little accumulation of leukocytes in this region of the lateral ventricle as compared to mice fed the Paigen diet (Figure 7B). The accumulation of leukocytes was found to extend ventrally to the choroid plexus of the ventricular space in mice fed a Paigen diet (Figure 7C), and mice fed a Western diet also show increased leukocyte infiltration in this ventral region, though numbers are reduced compared to the Paigen diet–fed mice (Figure 7D). Overall, the anti–IL-1β antibody reduced leukocyte accumulation in the ventral ventricle independently of diet, as compared to control antibody (*P* < 0.05, two-way ANOVA).

### Atherogenic Diet Results in Lipid Accumulation in the Brain, and IL-1β Neutralization Reduces Lipid-Associated Inflammation in the Lateral Ventricle

**Figure 8.**
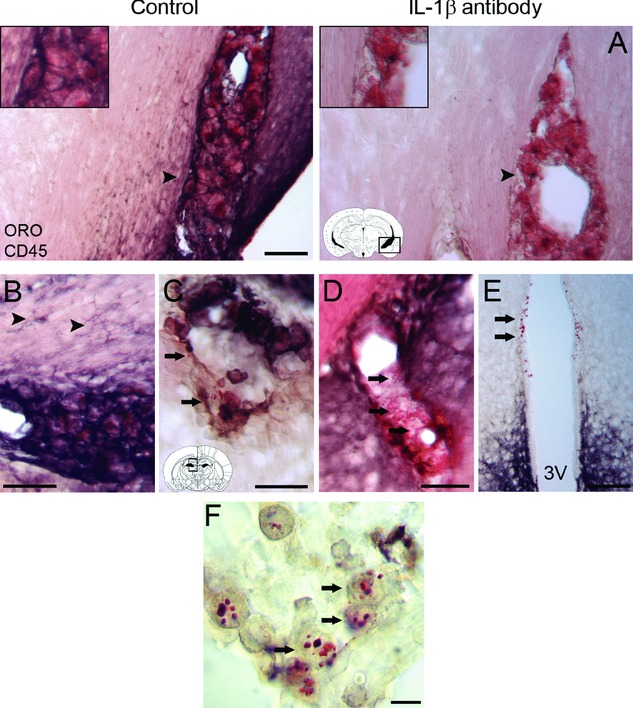
Atherogenic diet results in lipid accumulation in the brain, and IL-1β neutralization reduces lipid-associated inflammation in the lateral ventricle. Immunostaining for CD45 (dark brown/purple) followed by Oil Red O staining (lipids; red) was performed on brain sections from mice fed Paigen or Western diet. A, IL-1β antibody treatment reduces the recruitment of CD45-positive cells in the ventral (caudal) lateral ventricle and periventricular microglial activation but has no effect on lipid accumulation (insets show higher magnification of the areas marked by arrowheads). B, Lipid accumulation is observed in the rostral ventricle below the hippocampus, associated with hippocampal microglial activation (arrowheads). C, Inflammatory cells are found recruited to lipid droplets (arrows) around the ventricular ependyma in the dorsal thalamus in mice fed Paigen diet. Lipid deposition (arrows) is seen in the wall of ventricle-associated blood vessels (D) in animals fed a Paigen diet. E, Mice fed Western diet display lipid droplets (arrows) in the wall of the third (3V) ventricle and microglial activation in the arcuate nucleus. F, Lipid droplets are observed in choroid plexus cells (arrows) in mice fed a Paigen diet. Location of panels B through D and F in the brain is shown on schematic on panel C. n=7. Scale bars: 50 μm (A through D), 100 μm (E), and 10 μm (F).

We have previously reported that mice fed a Paigen diet display focal lipid deposition and inflammation in the brain parenchyma.^6^ In the present study, the number of focal pathologies was relatively low in the parenchyma and was not affected by IL-1β neutralization. However, Oil Red O staining revealed lipid accumulation in the lateral ventricle in mice fed a Paigen diet, which was associated with recruitment of CD45-positive cells and microglial activation (Figure 8A Through 8F). Lipid accumulation and inflammation were observed in the choroid plexus, the lumen of the dorsal (rostral) and ventral (caudal) parts of the lateral ventricle, and both the thalamic and hippocampal ventricular walls near the ependyma (Figure 8A Through 8C). Lipid deposition was frequently seen on the wall of larger ventricle-associated blood vessels (Figure 8D) and occasionally in small parenchymal arteries. Lipid droplets were found inside choroid plexus cells (Figure 8F) and in the subfornical organ, associated with the accumulation of CD45-positive cells. Anti–IL-1β antibody greatly reduced CD45-positive cells with leukocyte and microglial morphology in the caudal lateral ventricle (Figure 8A) but had no effect on lipid accumulation. Lipid deposition and inflammation were far less pronounced in the brains of mice fed a Western diet. Lipid droplets in the ependymal wall of the third ventricle were seen in mice fed both Paigen and Western diets (Figure 8F).

## Discussion

We show that central nervous system inflammatory pathology is associated with the development of atherosclerosis in high-fat diet–fed ApoE^−/−^ mice and, through genetic deletion of IL-1R1 or neutralization of IL-1β, provide evidence that this is mediated by IL-1. These findings identify IL-1 as a key driver in the development of cerebrovascular inflammation in response to atherogenic diet and systemic vascular disease.

Neutralization of IL-1β had antiinflammatory effects in peripheral organs. High-fat feeding increases hepatic inflammation, presumably because of an increased accumulation of cholesterol.^19^ Treatment of hepatic cells with IL-1β also raises the level of cholesterol uptake and accumulation.^20^ Therefore, the reduced hepatic inflammation seen in the present study could be due to the decreased availability of IL-1β to the hepatic cells and thus a decreased uptake of cholesterol.

Treatment with an anti–IL-1β antibody attenuated brain leukocyte accumulation in ApoE^−/−^ mice in the lateral ventricle, but this effect was confined to the ventral ventricular region in mice fed the less aggressive Western diet,^21^ with the latter showing a much lower brain leukocyte invasion than Paigen diet–fed animals. Vascular activation was reduced by neutralization of IL-1β in mice fed a Western diet, but only a trend toward reduction was seen in Paigen diet–fed animals (which had much higher number of VCAM-positive blood vessels), and no effect was seen on microglial activation in mice fed either diet. These observations are not fully consistent with the findings in ApoE^−/−^/IL-R1^−/−^ mice fed a Paigen diet and may be because of limited brain penetration of the anti-IL-1β antibody, or because IL-1α, which can also mediate activation of IL-1R1, contributes to changes in the brain and cerebrovasculature.

ApoE^−/−^ mice fed either a Western or Paigen diet are known to develop atheroma throughout the aorta.^22^ Treatment with an anti–IL-1β antibody caused a significant reduction in the atherosclerotic lesion area in the descending aorta in mice fed both a Paigen and Western diet and in the aortic root in mice fed a Paigen diet. These data are consistent with a recent study that reported similar effectiveness of another anti–IL-1β antibody treatment for atherosclerosis.^15^ Because ApoE^−/−^/IL-1R1^−/−^ mice fed an atherosclerotic diet also develop reduced peripheral pathology,^14^ it is not possible from the findings of this study to determine the specific site of IL-1 action in driving central nervous system pathology. Reversal of the effects of high-fat diet in mice lacking the IL-1 receptor may be due to the absence of IL-1 signaling within the cerebral vasculature or in the brain or may be secondary to the amelioration of peripheral pathology. The fact that there was no diet-dependent difference in atherosclerotic lesion size but Paigen diet induced more substantial cerebrovascular inflammation than did Western diet argues for some brain-specific effects of IL-1 in response to atherogenic diet and systemic vascular inflammation. Regardless of the site of IL-1 action, these data show benefits of IL-1 blockade on multiple tissues, notably the brain.

Central nervous system inflammation is observed in response to peripheral disease and may itself contribute to the development of vascular diseases. Recent experimental data indicate a role for centrally mediated signaling by inflammatory cytokines such as IL-1β and tumor necrosis factor-α in neurogenic hypertension or metabolic disease.^23–25^ Neutralization of IL-1β reversed weight loss in mice fed a Paigen diet, and the decline in voluntary physical activity in ApoE^−/−^ mice was also reversed by genetic deletion of the IL-1 receptor. Central IL-1–mediated effects on weight loss might be important in light of recent data that implicated inflammation-induced central IL-1 signaling in muscle atrophy, which was found to be dependent on the hypothalamic–pituitary–adrenal axis.^26^ Paigen diet induces more substantial proinflammatory changes than does the Western diet, which was also indicated by the higher levels of CD45-positive cells in the lateral ventricle and the reduction of body weight, as opposed to the body weight increase seen in Western diet–fed mice. The different mechanisms of action of Paigen and Western diets^21,22^ and, therefore, the different levels of systemic and central inflammation induced might also explain differences seen in the effect of IL-1β neutralization on the various inflammatory parameters we studied in the brain.

Lipid deposition in ventricle-associated blood vessels, choroid plexus, circumventricular organs, and ventricles indicates pronounced vascular effects elicited by an atherogenic diet in the brains of mice fed a Paigen diet. Lipid accumulation appeared to take place independently from IL-1β. However, recruitment of inflammatory cells was localized to lipid accumulation in the brain and was markedly reduced in response to IL-1β neutralization, which suggests that local lipid deposition–induced stimuli might be directly involved in inflammatory cellular responses in the brain. Collectively, our data suggest that interventions against central and systemic effects of vascular diseases could have therapeutic potential. Elevated systemic inflammatory burden is considered a high risk for cardiovascular and cerebrovascular diseases.^27–29^ Clear association exists between depression and cardiovascular/metabolic disease,^30–32^ and IL-1 signaling has been linked closely with the development of decreased motivational disorders such as depression.^33–35^

Therefore, patients with multiple risk factors for heart disease or stroke might benefit from neutralization of systemic IL-1β activity. Similarly, the potential effects of high-fat diet and atherosclerosis on cognitive decline and depression might be reduced by interventions against IL-1 signaling.

## Conclusions

We present evidence that the cytokine IL-1 contributes to neuroinflammatory changes in mice developing diet-induced atherosclerosis. IL-1β blockade provides a potential therapeutic opportunity to limit atherosclerosis, associated neuroinflammation, and hence cerebrovascular disease such as stroke and vascular dementia.
